# Bladder Neck Contracture after Transurethral Resection of the Prostate for Benign Prostatic Hyperplasia Treated with a Thermo-Expandable Metal Stent (Memokath® 045)

**DOI:** 10.1155/2018/2439421

**Published:** 2018-05-09

**Authors:** Jan Wen, Bettina Nørby, Palle Jörn Sloth Osther

**Affiliations:** ^1^Urological Research Center, Department of Urology, Vejle Hospital, Vejle, Denmark; ^2^Institute of Regional Health Research, University of Southern Denmark, Vejle, Denmark

## Abstract

Bladder neck contracture following transurethral resection of the prostate is a rare but feared complication. Treatment is often challenging with significant recurrence rates. In this report, we present a complicated case treated with a simple procedure. A 75-year-old male developed urinary retention due to bladder neck contracture after transurethral resection of the prostate. He was initially treated with several transurethral incisions, but the obstruction recurred few months after each incision. At urethroscopy, the bladder neck was completely obstructed. Using both retrograde and antegrade endoscopy, it was possible to place a through-and-through guidewire, after which the length of the stricture could be measured. Subsequently, the stricture was slightly dilated, and a double-cone thermo-expandable metal stent (Memokath 045) could be placed. The correct position was monitored with antegrade and retrograde endoscopy, securing the proximal cone expanded above the stricture and the distal cone above the sphincter. The patient was discharged the same day with spontaneous voiding and minimal residual urine. Twenty-one months after stent placement, the patient still had no complaints of his urination. Thus, the double-cone thermo-expandable metal stent, Memokath 045, may be a durable option for treatment of complicated bladder neck contracture after TURP for benign prostatic hyperplasia.

## 1. Introduction

Bladder outlet obstruction (BOO) as a result of benign prostatic enlargement (BPE) is often related to lower urinary tract symptoms (LUTS) [1]. Complications such as detrusor failure, renal failure, recurrent urinary tract infections, urinary retention, hematuria, and bladder stones are associated with untreated BOO [2].

Transurethral resection of the prostate (TURP) has been considered the gold standard surgical treatment for BPE for decades, with success rates up to 90% [3]. The most common complications include bleeding, infection, retrograde ejaculation, urethral stricture, and incontinence [4, 5]. Additionally, TURP may be complicated by bladder neck contracture (BNC), with a reported frequency of 0–4.9% [6–8]. BNC typically occurs within two years after surgery [9]. Despite the limited knowledge about the pathophysiology of BNC, the incidence probably depends on the surgical technique employed [10, 11], and potential risk factors have been proposed to be a low adenoma weight, extensive resection of the bladder neck, and use of a large resecting loop [6, 9, 12, 13]. Furthermore, a smoking history and a history of more than two previous endoscopic BNC procedures are associated with a poor prognosis [14].

Treatment of BNC is challenging with no clear guideline present [13, 15]. Therefore, studies of existing techniques are needed [16]. In this report, we present a challenging case treated successfully with a simple procedure.

## 2. Case Presentation

In 2013, a 75-year-old Caucasian male underwent evaluation for LUTS at another hospital. Residual urine of 500 ml was found. A cystoscopy revealed a large bladder diverticulum. Urodynamic evaluation showed BOO, and a prostate volume of 32 cc was found by transrectal ultrasonography. The patient had no comorbidity, and BMI was normal.

The patient initially performed clean intermittent catheterization (CIC) three times a day. Due to persistent residual urine of approximately 500 ml and patient's dissatisfaction with CIC, a TURP was performed in December 2013 with resection of 7 g of tissue. The bladder diverticulum was left untouched. The surgery was uneventful, and the patient was discharged after the procedure with no complications. In the following weeks, the patient experienced significant improvements in flow and voided volume.

Two months later, the symptoms recurred, and the patient developed urinary retention. Cystoscopy showed almost completely obstruction at the bladder neck. In May 2014, a bladder neck incision was performed, but, only one month later, another incident of urinary retention occurred. A total of three transurethral resections/incisions of the bladder neck were done before placing a suprapubic catheter as a permanent solution.

In 2015, the patient was referred to our department due to dissatisfaction with the suprapubic catheter. The initial plan was to treat the patient with balloon dilation and deep lateral incisions at the 3 and 9 o'clock positions, a procedure that has been reported with an overall success rate of 86% [14]. The procedure, however, was impossible to perform, due to a completely closed bladder neck.

After mutual consent between patient and surgeons, we decided to treat him with a metal stent due to considerations regarding pros and cons with different reported surgical treatments.

**Figure 1:**
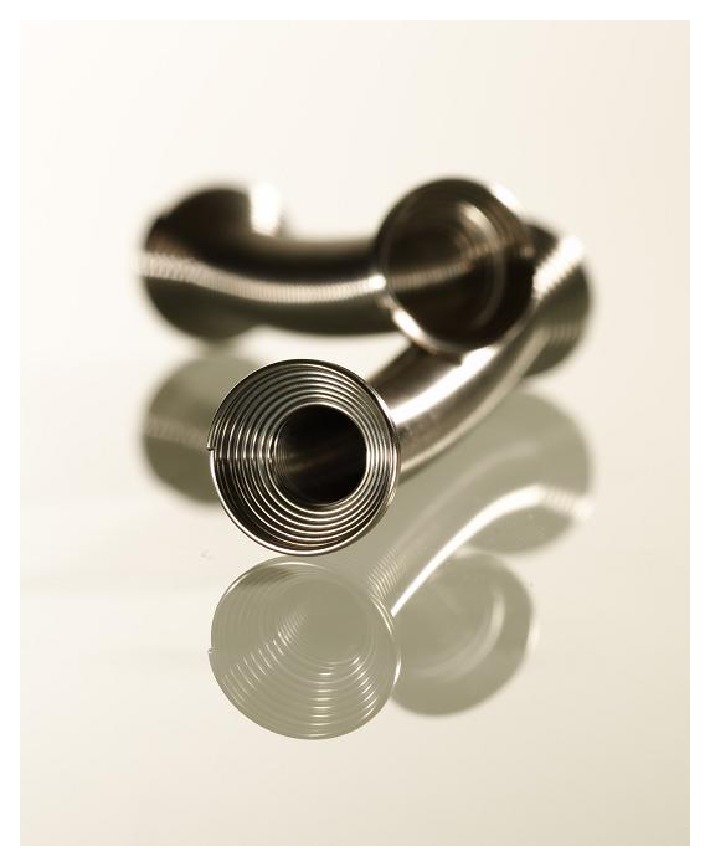
Thermo-expanded Memokath 045.

**Figure 2:**
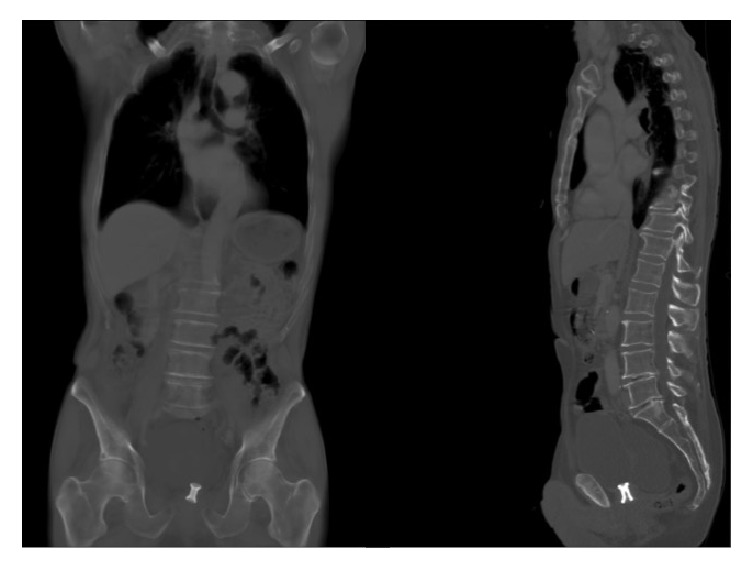
Frontal and lateral view of a CT (bone window) showing the double-cone Memokath 045 placed at the bladder neck.

Three months later, in April 2016, we successfully placed a thermo-expandable metal stent (Memokath 045 PNN Medical, Copenhagen, Denmark (Figure 1)) in the bladder neck. A small passage between urethra and the bladder was identified by simultaneous urethroscopy and antegrade cystoscopy through the suprapubic canal. A through-and-through guidewire could then be placed, and the length of the stricture (0.8 mm) measured using a dual lumen ureteral catheter (COOK Medical, Bloomington, USA). Subsequently, the passage was slightly dilated, after which a double expanding Memokath 045 could be placed and thermo-expanded (Figure 2), endoscopically controlled from above and below, ensuring that the proximal cone expanded above the stricture and the distal cone was placed above the sphincter. The patient was discharged with spontaneous micturition the same day and minimal residual urine.

Four months later, in August 2016, the patient showed satisfying results in urine flow examination, and ultrasonography of the bladder showed an acceptable residual urinary volume of 150 ml. Twenty-one months later (January 2018) the patient still had no complaints of his urination.

## 3. Discussion

TURP was probably not the ideal solution for this patient with a small volume prostate, and this may have been the reason for BNC development. Transurethral incision of the prostate (TUIP) might have been more appropriate [17]. Also, three bladder neck incisions prior to stent placement must be considered inappropriate.

BNC is associated with significant morbidity and represents a major clinical challenge, which is reflected by various treatment modalities presented in the literature, such as balloon dilatation, deep lateral incisions (Mercedes) using cold knife as well as laser, bipolar plasma vaporization, bladder neck incision with intralesional injection of Mitomycin C, and Y-V and T-plasty [9–14]. All treatment modalities are associated with a significant recurrence rate, as exemplified by the present case.

Memokath is a nickel-titanium alloy coil with shape memory [18]. It expands at 60°C and uncoils at 5°C and is resistant to compression. Due to the stents uncoiling in cold water, they are very easy to remove, even if they are incrusted [18]. Specific subtypes of Memokath stents have been developed for ureteral strictures, BPE, and urethral strictures [18], and Memokath stents have even been used for treatment of ureteral avulsion [19], ureteroileal anastomotic stricture [20], strictures in renal transplant kidneys [21], and ureterovaginal fistulas [22]. Regarding bulbar urethral strictures treated by Memokath, data in the literature are conflicting. Jordan et al. reported data from a randomized trial in patients with recurrent bulbar urethral strictures and showed that patients treated with dilation or urethrotomy and a Memokath stent 044TW maintained urethral patency significantly longer than those treated with dilation or urethrotomy alone [23]. However, a recent investigative pilot stage 2A study in 16 males was unable to confirm this [24].

In many of the above-mentioned indications, Memokath stents represented a simple minimal invasive endourological procedure for traditional major surgical dilemmas, as was the case with the present BNC. Considering the patient had no comorbidity, he could probably have undergone a more extensive and definite treatment. However, with no clear guidelines and great disease diversity, treatment choice always has to be personalized. In this scenario the Memokath 045 offers a minimal invasive solution.

The key to success in the present case was (1) a simultaneous retrograde and antegrade approach with through-and-through guidewire, securing measuring the right length of the stricture and visually securing stent placement with the upper cone in the bladder and the lower cone above the sphincter and (2) minimal dilation for firm attachment of the stent during thermo-expansion. As with other metal stents, patients should undergo regular follow-up since the stents may encrustate and migrate [25]. Spontaneous resolution of ureteral strictures after Memokath® placement has been reported [25]; however, in the case of BNC, spontaneous resolution probably cannot be expected, and if removal of the stent is necessary due to malfunction, this will most likely require replacement of a new stent or another therapeutic approach.

In conclusion, the double-cone thermo-expandable metal stent, Memokath 045, may be a durable option for treatment of complicated bladder neck contracture after TURP for benign prostatic hyperplasia, using both antegrade and retrograde endoscopy for monitoring correct stent placement.
